# Functional Role of the Disulfide Isomerase ERp57 in Axonal Regeneration

**DOI:** 10.1371/journal.pone.0136620

**Published:** 2015-09-11

**Authors:** Valentina Castillo, Maritza Oñate, Ute Woehlbier, Pablo Rozas, Catherine Andreu, Danilo Medinas, Pamela Valdés, Fabiola Osorio, Gabriela Mercado, René L. Vidal, Bredford Kerr, Felipe A. Court, Claudio Hetz

**Affiliations:** 1 Biomedical Neuroscience Institute, Faculty of Medicine, University of Chile, Santiago, Chile; 2 Program of Cellular and Molecular Biology, Center for Molecular Studies of the Cell, Institute of Biomedical Sciences, University of Chile, Santiago, Chile; 3 Millenium Nucleus for Regenerative Biology, Faculty of Biology, Pontificia Universidad Católica de Chile, Santiago, Chile; 4 Program of Immunology, Institute of Biomedical Sciences, University of Chile, Santiago, Chile; 5 Neurounion Biomedical Foundation, CENPAR, Santiago, Chile; 6 Centro de Estudios Científicos, Valdivia, Chile; 7 Department of Immunology and Infectious diseases, Harvard School of Public Health, Boston MA, United States of America; University of Edinburgh, UNITED KINGDOM

## Abstract

ERp57 (also known as grp58 and PDIA3) is a protein disulfide isomerase that catalyzes disulfide bonds formation of glycoproteins as part of the calnexin and calreticulin cycle. ERp57 is markedly upregulated in most common neurodegenerative diseases downstream of the endoplasmic reticulum (ER) stress response. Despite accumulating correlative evidence supporting a neuroprotective role of ERp57, the contribution of this foldase to the physiology of the nervous system remains unknown. Here we developed a transgenic mouse model that overexpresses ERp57 in the nervous system under the control of the prion promoter. We analyzed the susceptibility of ERp57 transgenic mice to undergo neurodegeneration. Unexpectedly, ERp57 overexpression did not affect dopaminergic neuron loss and striatal denervation after injection of a Parkinson’s disease-inducing neurotoxin. In sharp contrast, ERp57 transgenic animals presented enhanced locomotor recovery after mechanical injury to the sciatic nerve. These protective effects were associated with enhanced myelin removal, macrophage infiltration and axonal regeneration. Our results suggest that ERp57 specifically contributes to peripheral nerve regeneration, whereas its activity is dispensable for the survival of a specific neuronal population of the central nervous system. These results demonstrate for the first time a functional role of a component of the ER proteostasis network in peripheral nerve regeneration.

## Introduction

The accumulation of abnormal protein aggregates in the form of oligomers and large inclusions is the hallmark of several neurodegenerative diseases including Alzheimer’s disease (AD), Parkinson’s disease (PD), amyotrophic lateral sclerosis (ALS), among other brain pathologies; and are now classified as protein misfolding disorders (PMDs) [1]. Alteration to the proteostasis network is a salient feature of most PMDs, where we highlight perturbations to the function of the endoplasmic reticulum (ER) as an emerging driver of neurodegeneration [2]. Around one third of the proteome is synthesized and folded at the ER, where a complex network of resident chaperones, foldases, quality control mechanisms, and co-factors ensure the correct folding of proteins to prevent abnormal aggregation and proteotoxicity [3]. Many conditions can alter the protein folding status of the ER, generating a condition known as ER stress [4]. To cope with ER stress cells activate the unfolded protein response (UPR) as an adaptive reaction to modulate the expression of hundreds of genes involved in almost every aspect of the secretory pathway [5, 6]. Protein disulfide isomerases (PDIs) represent a group of well-known UPR-target genes induced in the nervous system under pathological conditions. Members of the PDI family are often upregulated in tissues derived from patients affected with PMDs, in addition to mouse models of the disease (reviewed in [7]). However, most evidence linking the biology of PDIs with neurodegeneration remains highly correlative and only a few functional reports are available in cell culture models.

One of the most studied PDIs is ERp57 (also known as Grp58 or PDIA3). ERp57 is a multifunctional protein located mostly at the ER lumen where it operates as a foldase and chaperone [8]. As a component of the calnexin (CNX) and calreticulin (CRT) cycle, ERp57 is predicted to participate in the folding of numerous cysteine-rich glycoproteins [9]. ERp57 can also function as a molecular chaperone preventing the formation of protein aggregates [10–13]. Besides, alternative roles of ERp57 are described beyond assisting protein folding, including the regulation of cell signaling, assembly of MHC complexes as a scaffold, and the regulation of apoptosis [7, 13, 14]. Accumulating evidence highlights the possible contribution of ERp57 to neurodegenerative diseases. For example, a proteomic study of brain samples derived from patients affected with a Prion-related disorder indicated that ERp57 is one of the most upregulated proteins [15]. We confirmed these findings and further validated the upregulation of ERp57 in animal models of the disease [16]. We also described that targeting ERp57 function in cell culture models revealed a neuroprotective activity against misfolded prions [17]. ERp57, and its closest homologue PDIA1, are also upregulated in the spinal cord from sporadic ALS cases [18, 19]. Consistent with these findings, proteomic analyses of spinal cord from an ALS mouse model revealed that ERp57 and PDIA1 are among the strongest induced proteins in symptomatic animals [20, 21]. Remarkably, PDIA1 and ERp57 were also identified as possible biomarkers to monitor disease progression in blood samples from ALS cases [22]. In addition, inactivation of PDIA1 by S-nitrosylation is observed in postmortem tissue derived from patients affected with ALS, PD and AD; a posttranslational modification that may ablate its neuroprotective activity [23, 24]. Moreover, we recently identified mutations in the genes encoding ERp57 and PDIA1 in ALS cases [25]. Intronic variants of *Pdia1* were also proposed as a risk factor to develop ALS [26]. In contrast, another report suggested a proapoptotic role of PDIA1 and ERp57 in models of Huntington’s disease and AD [27]. Despite all this evidence linking PDIs to neurodegenerative conditions, the specific contribution of these foldases to the diseases process *in vivo* still remains elusive.

Only a few studies have evaluated the contribution of the ER chaperone network to the biology of the nervous system. CNX deficient animals develop motor function impairment, which is caused by the loss of large and medium myelinated nerve fibers [28]. This phenomenon results in demyelination and reduced nerve conduction velocity in the sciatic nerve [29]. In the context of ALS, deletion of one copy of the CRT gene accelerates the progression of the disease, possibly involving muscle denervation and enhanced protein aggregation in the spinal cord [30]. In addition, mutation in SIL1, a cofactor of BiP, results in spontaneous degeneration of cerebellar mutant Purkinje cells [31]. SIL1 was recently shown to protect motoneurons from degeneration on an ALS mouse model [32]. Furthermore, a knock-in mutant mouse model to inactivate BiP was generated, where heterozygous animals showed a normal life span but developed serious motor problems and neurodegeneration during aging [33]. Although these reports have demonstrated the relevance of the ER proteostasis network to the physiology of the nervous system and neurodegeneration, no studies are available defining the therapeutic potential of PDIs *in vivo*. To address this question, here we generated a neural-specific ERp57 transgenic mouse (Tg-ERp57) to explore the possible contribution of this central PDI to neurodegenerative processes. Remarkably, Tg-ERp57 mice displayed accelerated locomotor recovery after peripheral nerve injury associated with improved axonal regeneration. In contrast, overexpression of ERp57 had no functional consequences on the survival of dopaminergic neurons in animals treated with a PD-inducing neurotoxin. Our results suggest a selective impact of ERp57 in maintaining protein homeostasis in the peripheral nervous system.

## Results

### Generation of an ERp57 transgenic mouse model in the nervous system

**Fig 1:**
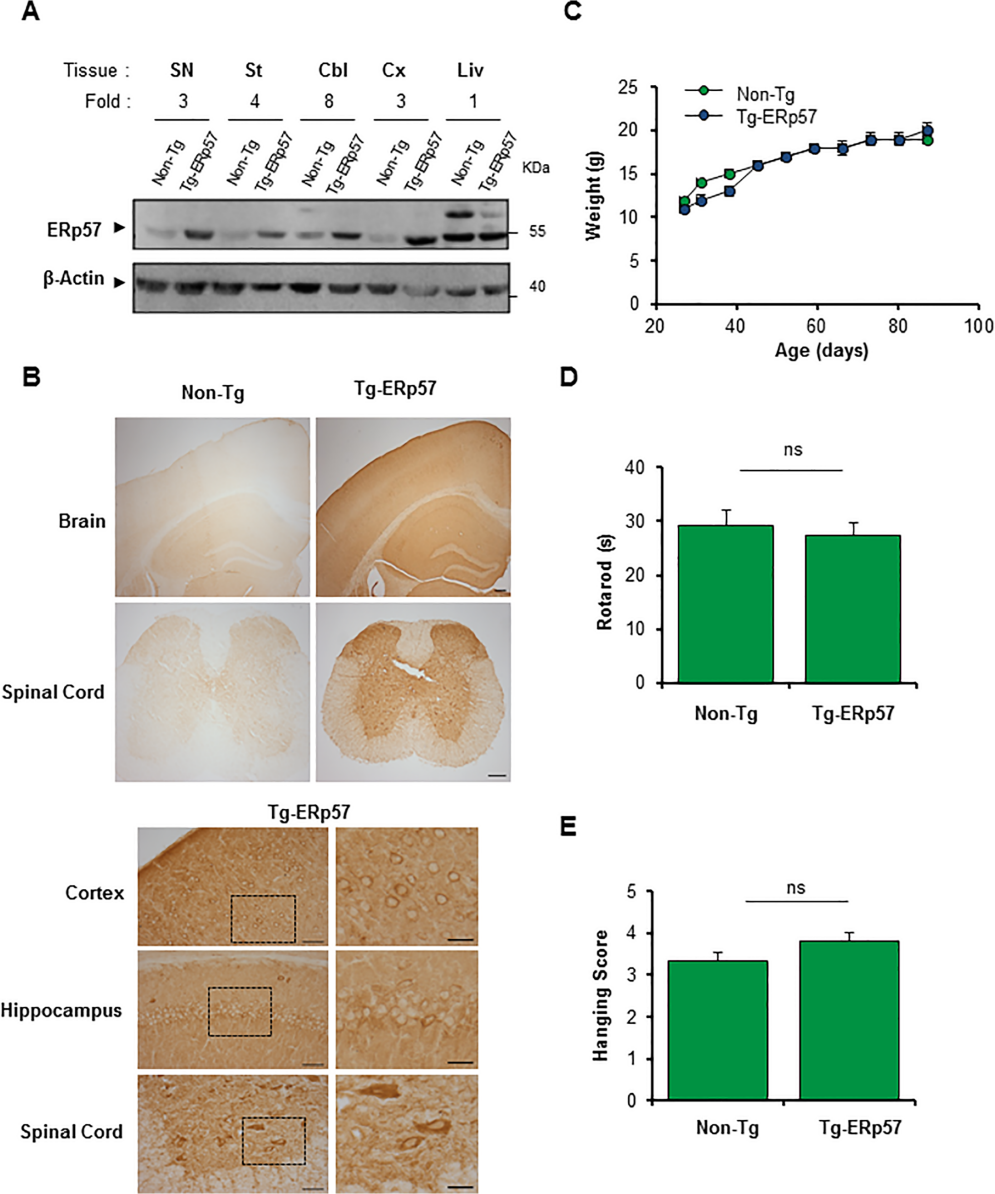
Generation of an ERp57 transgenic mouse model. (A) ERp57 protein levels were analyzed in nervous system tissue of a neuronal specific ERp57 transgenic (Tg-ERp57) mice and littermate control non-transgenic (Non-Tg) animals using Western blot. The following tissue was analyzed: substantia nigra (SN), striatum (St), cerebellum (Cbl), cortex (Cx) and liver (Liv). β-Actin was used as a loading control. In the upper panel, quantification of the relative ERp57 levels is presented normalized to Non-Tg animals. (B) Immunohistochemistry anti-FLAG was performed in nervous system tissue of Tg-ERp57 mice and littermate control non-transgenic (Non-Tg). The following tissue was analyzed: cortex, hippocampus, and spinal cord ventral horn. At the right panels, magnification of indicated areas is shown. Scale bar: 200 μm for lower magnifications and 50 μm (left) and 25 μm (right) for higher magnification. (C) Analysis of body weight in Tg-ERp57 and Non-Tg animals over time. (D) Rotarod performance was measured every week starting at 50 days of age in animals presented in C. (E) Hanging test was performed every week starting at approximately at 50 days of age as described in material and methods. In C-D mean ± SEM is presented of each group. Tg-ERp57 (n = 15) and Non-Tg (n = 18) animals. Student’s unpaired t-test was performed to calculate statistical significance (*: *p* < 0.05; n.s.: non-significant).

To investigate the possible role of ERp57 in the nervous system we generated a transgenic mouse model that overexpresses the human *Erp57/Pdia3* cDNA under the control of the PrP promoter (termed Tg-ERp57) to express the protein at high levels in the nervous system. The exogenous human ERp57 was designed to carry a FLAG-tag at the C-terminus for detection purposes. Several founder lines were generated, however only one line was used in the present study because it showed the highest expression in spinal cord, peripheral nerve, and brain tissue that was maintained over several generations. Routinely, Tg-ERp57 heterozygous animals were crossed with C57BL/6 mice to obtain transgenic and non-transgenic offspring in B6 background with 98% purity. Animals were viable and born in a Mendelian ratio (Table 1). To confirm the overexpression of ERp57 in the nervous system, we dissected tissue from substantia nigra (SN), striatum, cerebellum, cortex, as well as the liver as negative control. An increase in ERp57 expression was observed in all tissues of the nervous system to different extends as determined by real-time PCR and Western blot analysis (Fig 1A, S1A and S1B Fig). Quantification revealed at least a three-fold increase in ERp57 protein levels in transgenic animals compared to littermate control mice (Fig 1A). We determined the distribution of ERp57-FLAG in different regions of the nervous system including cortex, hippocampus and spinal cord using an anti-FLAG antibody by immunohistochemistry (Fig 1B). A neuronal-staining pattern was observed in Tg-ERp57 mice in all CNS tissues based on morphological analysis. The staining was cytosolic and excluded the nucleus consistent with the known subcellular distribution of ERp57 (Fig 1B). Co-immunofluorescence analysis of brain tissue indicated that ERp57-FLAG expression was mostly restricted to the neuronal compartment and not astrocytes (S1C Fig). Body weight measurements were performed once a week starting at 30 days of age. Normal growth curves were obtained for Tg-ERp57 animals (Fig 1C). Furthermore, evaluation of the motor performance of Tg-ERp57 mice using the rotarod assay showed no alterations to this parameter (Fig 1D). Similarly, coordination and muscle strength measurements using the hanging test did not reveal any gross effects after ERp57 overexpression *in vivo* (Fig 1E).

**Table 1:** Ratio of births of ERp57 transgenic mice.

Animals	Non-Tg	Tg-ERp57	Total
**N° Observed**	54	58	112
**Expected (%)**	50	50	100
**Observed (%)**	48.2	51.8	100

Genotypic distribution (percentage) of the offspring obtained from crosses, carried out to amplify the colony under study (see Materials and Methods). Non-Tg: ERp57 non-transgenic mice Tg-ERp57: ERp57 transgenic mice. The data correspond to generation 3 to 6 of the colony.

### ERp57 overexpression does not affect degeneration of dopaminergic neurons

**Fig 2:**
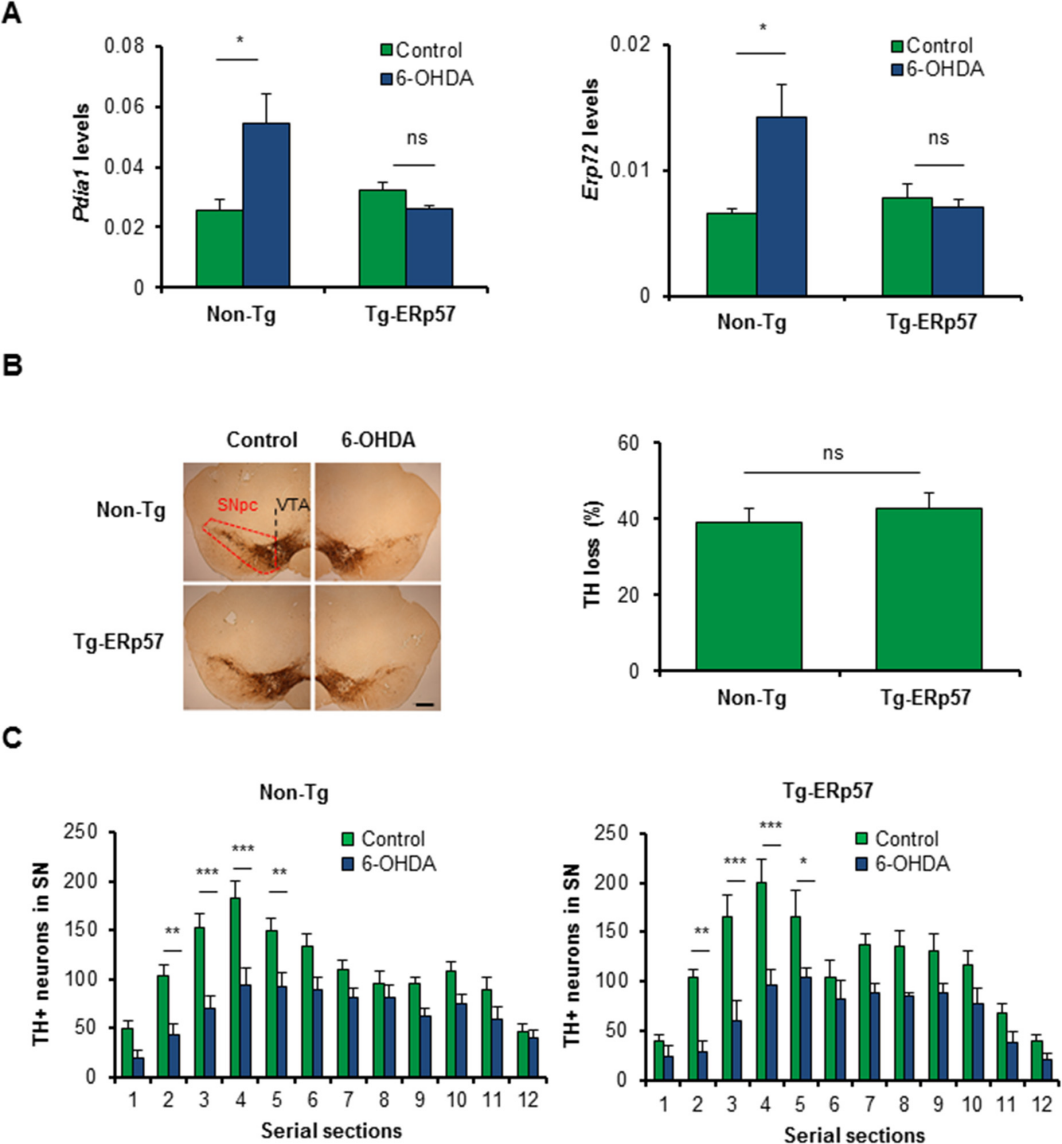
Effects of ERp57 overexpression on the survival of dopaminergic neurons after exposure to 6-OHDA. (A) *Pdia1* and *Erp72* mRNA levels were determined in dissected SNpc of Non-Tg and Tg-ERp57 mice 7 d after 6-OHDA injection. Injected and non-injected sides were analyzed using total cDNA and real-time PCR (n = 3 per group). Data is presented as mean and SEM. (B) Non-Tg and Tg-ERp57 mice were injected with 8 μg of 6-OHDA in the right striatum, and after 7d dopaminergic neurons (TH^+^) were quantified by anti-TH immunohistochemistry. Total content of TH-positive somas was measured in midbrain sections covering the entire SN, in the non-injected (control) and injected (6-OHDA) side, for the indicated genotype (n = 8, Non-Tg; n = 5, Tg-ERp57). Scale bar: 200 μm. (C) Histograms show the number of TH-positive neurons of injected and non-injected sides in 25 μm midbrain serial sections separated by 100 μm and covering the entire SNpc. The numbers of serial sections indicate the orientation from anterior to posterior. Statistical analysis was performed using Mann-Whitney test for all quantifications except for (C) where two-way ANOVA was used followed by Bonferroni posttest (*: *p* < 0.05; **: *p* < 0.01; ***: *p* < 0.001. n.s.: not significant.).

ER stress is a well-validated pathological mechanism underlying the loss of dopaminergic neurons in PD [34]. Genetic or pharmacological manipulation of the UPR has functional effects on neurotoxin-based models of PD [35–38]. Since alterations in the activity of PDIs is observed in the brain of PD patients and animal models of the disease [24, 39, 40], we evaluated the possible effects of ERp57 overexpression in dopaminergic neuron survival using a pharmacological model of PD. Quantification of ERp57 levels at the SNpc of Tg-ERp57 mice indicated a near 4-fold overexpression levels (S1A Fig). We performed stereotaxic injection of the neurotoxin 6-hydroxydopamine (6-OHDA) into the striatum of Tg-ERp57 and non-transgenic (Non-Tg) animals to induce the progressive degeneration of the nigro-striatal circuit. Seven days post-surgery, we analyzed the neurodegenerative process at the SNpc by monitoring dopaminergic neuron loss after tyrosine hydroxylase (TH) immunohistochemistry (S2A and S2B Fig). We first determined the impact of ERp57 in the upregulation of PDIs in animals challenged with 6-OHDA. We determined the mRNA levels of two PDI family members, PDIA1 and ERp72 in dissected midbrain regions, and observed a significant increase in Non-Tg animals injected with 6-OHDA (Fig 2A). Remarkably, we found a full inhibition on the upregulation of these two ER foldases in Tg-ERp57 animals (Fig 2A), suggesting that the levels of ERp57 overexpression obtained in our model have functional protective effects on reducing stress responses at the SNpc.

We then performed quantification of the number of dopaminergic neurons in serial sections of the entire SNpc region. Surprisingly, administration of 6-OHDA led to an equivalent loss of dopaminergic neurons of around 40% in both Tg-ERp57 and Non-Tg mice (Fig 2B and S2C Fig). We recently described that deficiency of the UPR transcription factor XBP1 affects the death of a subset of dopaminergic neurons of the SNpc [38]. To address this issue in Tg-ERp57 mice, we performed a quantitative spatial distribution analysis of dopaminergic neurons of serial sections of the entire SNpc. Again, we found no differences in the distribution of TH-positive dopaminergic neurons neither at basal level nor after 6-OHDA injections in both genotypes (Fig 2C). Thus, although ERp57 overexpression reduces stress levels after 6-OHDA injections, it does not prevent neuronal loss at the SNpc.

### ERp57 overexpression does not affect striatal denervation and motor control

**Fig 3:**
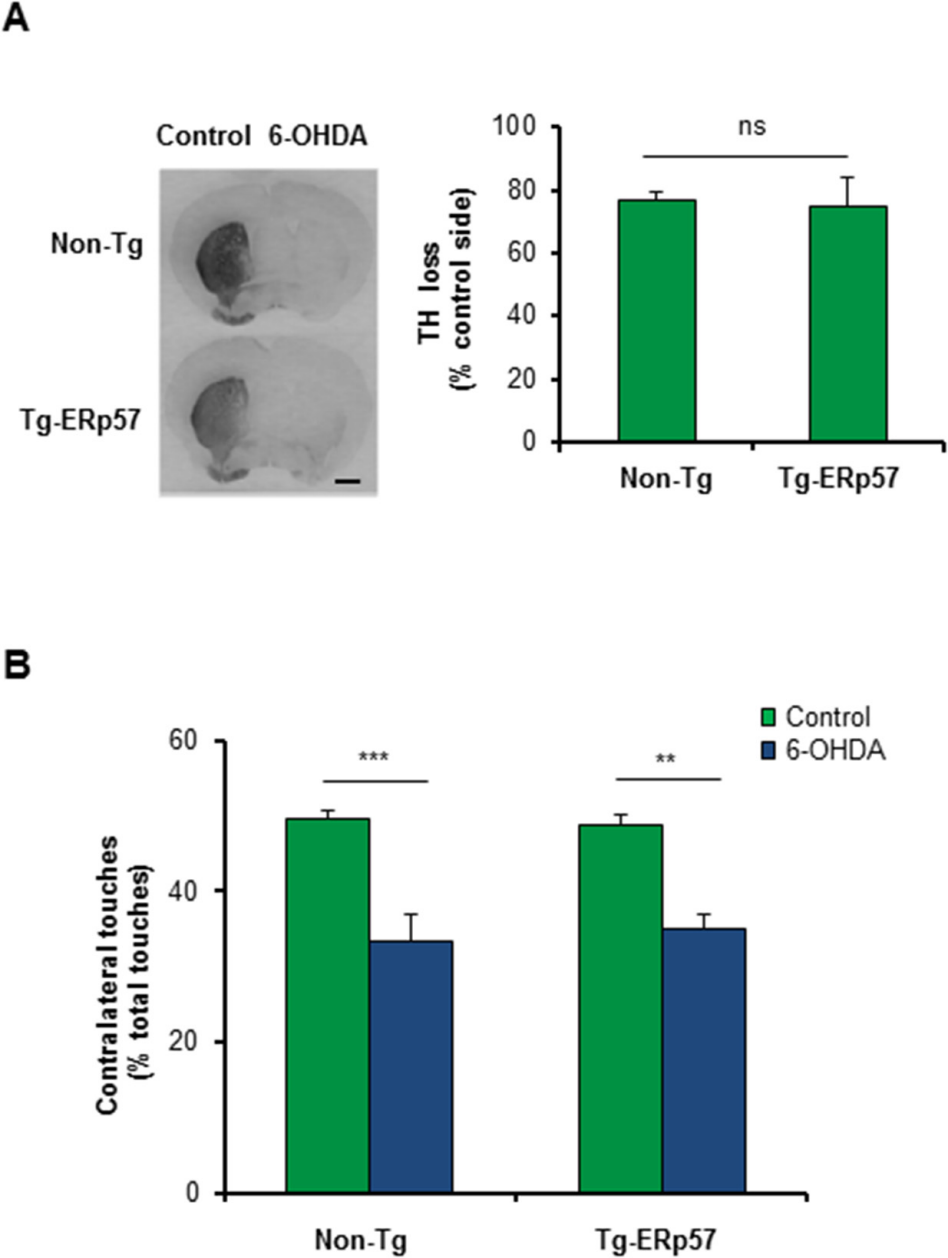
ERp57 overexpression does not affect 6-OHDA-induced striatal denervation or motor control. (A) Immunohistochemistry analysis was performed in striatal sections to quantify 6-OHDA–induced denervation in both injected (6-OHDA) and non-injected (control) sides. Scale bar: 1 mm. Right panel: The integrated density of pixel intensity was calculated from images of anti-TH immunohistochemistry covering the entire striatum and expressed as a percentage of TH loss relative to the control side (n = 8, Non-Tg; n = 5, Tg-ERp57). Scale bar: 200 μm. (B) Cylinder test was performed to evaluate spontaneous motor changes associated with dopamine depletion in the striatum of 6-OHDA injected mice. The cylinder test consist in to put the animals in a glass and record the number times the mouse touch the glass wall with each forepaw. Non-Tg (n = 10) and Tg-ERp57 (n = 8). Data is presented as mean and SEM. Statistical analyses were performed using Mann-Whitney test (A) and the Student t test (B). (*: *p* < 0.05; **: *p* < 0.01; ***: *p* < 0.001. n.s.: not significant.)

An early event observed in PD is axonal degeneration, leading to the loss of innervation of striatal neurons before dopaminergic neuron death [41]. We therefore determined the extent of striatal denervation triggered by 6-OHDA administration in Tg-ERp57 mice. We quantified TH staining of the whole striatum using serial sections (S2B Fig), and observed that the percentage of TH loss in the striatum was similar in the Tg-ERp57 and Non-Tg groups, reaching a reduction of near 80% of TH staining (Fig 3A). To evaluate the functionality of the nigro-striatal circuit we performed the cylinder test. This assay measures the spontaneous motor changes triggered by dopamine depletion in the striatum, associated to an asymmetrical use of both forepaws (see methods). Analysis of this behavioral test revealed no differences between Tg-ERp57 and control animals challenged with 6-OHDA (Fig 3B). Thus, the overexpression of ERp57 does not protect dopaminergic neurons against the PD-triggering neurotoxin 6-OHDA.

Based on these negative results, we decided to explore whereas 6-OHDA triggers a classical ER stress response. We monitored the expression levels of *Chop* using immunohistochemistry. In contrast to previous findings [42], we did not observe an upregulation of CHOP at the SNpc in both Tg-ERp57 and Non-Tg animals (S3A Fig, upper panel). As positive control we injected the SNpc with tunicamycin, a pharmacological inducer of ER stress (S3A Fig, bottom panel). We complemented this analysis by monitoring *Chop* mRNA levels by real time PCR of dissected midbrain tissue, obtaining virtually identical results (S3B Fig). We also measured the levels of *Xbp1* mRNA splicing in the striatum and SNpc of 6-OHDA injected animals using RT-PCR in time course experiments. Again, no signs of UPR activation were observed (S3C and S3D Fig). Since ER stress has been extensively reported in neuronal cell lines treated with PD-inducing neurotoxins, we exposed SH-SY5Y human dopaminergic neurons to different concentrations of 6-OHDA, in addition to perform kinetic analysis. In contrast to our previous results *in vivo*, treatment of cells with 6-OHDA induced a robust but transient activation of *Xbp1* mRNA splicing (S3E Fig). This extensive characterization suggests that 6-OHDA modulates the expression of PDIs at the SNpc in the absence of a global ER stress response.

### Overexpression of ERp57 accelerates functional recovery after peripheral nerve injury

**Fig 4:**
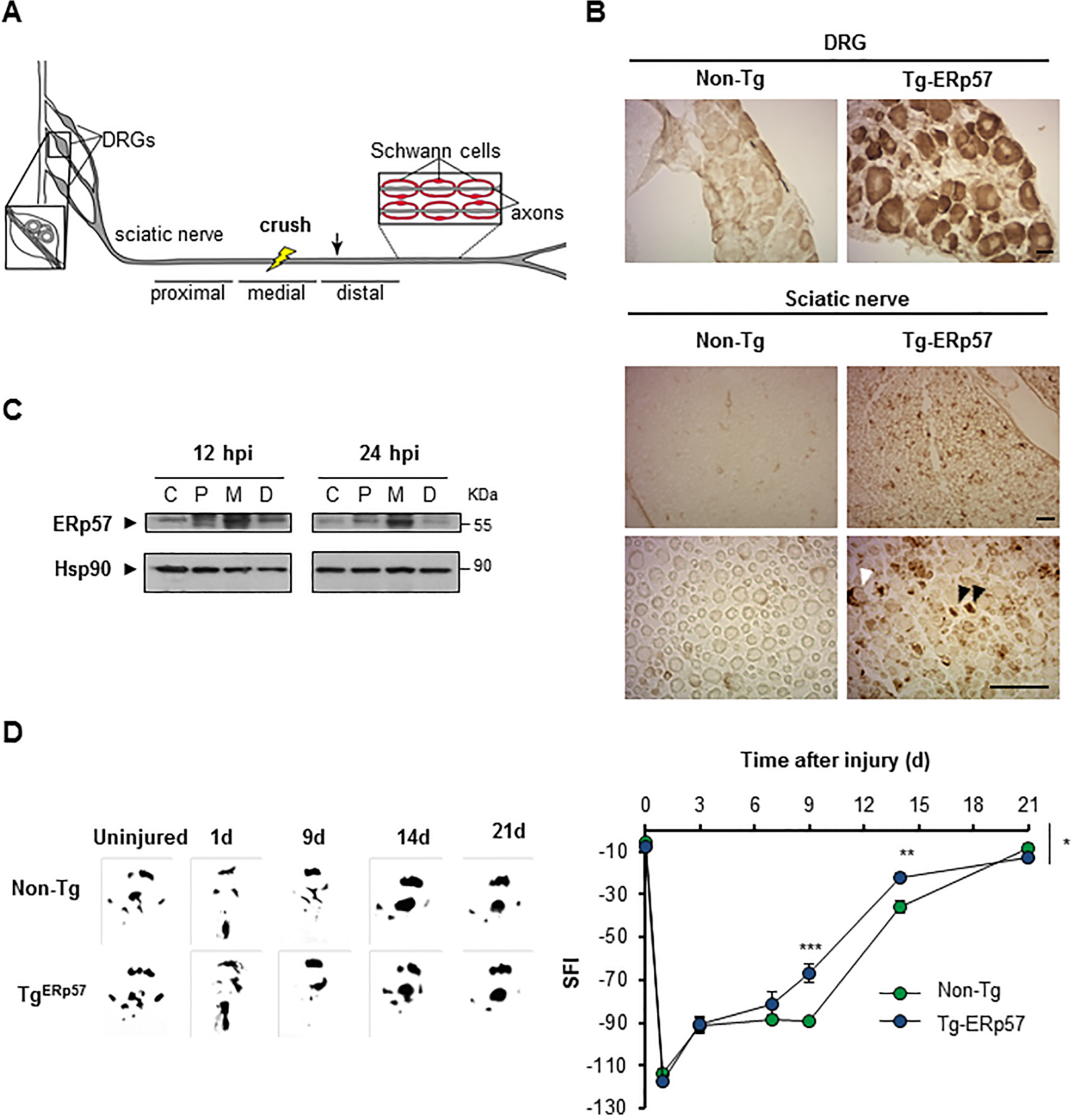
Overexpression of ERp57 in transgenic mice enhances locomotor recovery after peripheral nerve injury. (A) Scheme of a peripheral nerve: Sciatic nerve is formed by the spinal nerves of the dorsal root ganglia (DRG) from the L3, L4 and L5 vertebrae. DRG contains somas of sensory neurons and axons from motoneurons located in the ventral horn of spinal cord. Schwann cells surround peripheral axons to form myelinated and unmyelinated fibers. Nerves were damaged by mechanical crush (yellow bolt) and a 5 mm region from the injury region and adjacent proximal and distal regions were removed for biochemical analysis. A 3 mm region distal to the injury was removed for histological analysis (arrow). (B) Immunohistochemistry anti-FLAG was performed in DRG and sciatic nerve (Sci) of Tg-ERp57 mice and littermate control non-transgenic (Non-Tg). Black and white arrowheads points immunoreactive axons and Schwann cells, respectively. Scale bar: 40 μm. (C) Wild-type mice were damaged in the right sciatic nerve and ERp57 expression was evaluated at 12 and 24 h post injury (hpi) in proximal, P, medial, M and distal, D region. Contralateral sham-operated nerves, C were used as controls. Images were obtained from the analysis of the same gel and membrane. Hsp90 expression was used as loading control. (D) Non-Tg and Tg-ERp57 mice (n = 7 animals per group) were injured and the locomotor performance was analyzed using the sciatic nerve functional index (SFI). (Left panel) SFI was measured for 21 days after injury. (Right panel) Representative footprints of the damaged hind limb of Non-Tg and Tg-ERp57 mice at 0 (uninjured), 1, 9, 14 and 21 days post-injury. Data are shown as mean ± S.E.M. * *p* < 0.05, ** *p* < 0.01, *** *p* < 0.001. For SFI analysis data were analyzed by two-way ANOVA followed by Bonferroni post test.

Recent reports indicate that ER proteostasis is also altered when the peripheral nervous system (PNS) is damaged after mechanical injury or due to neurodegenerative conditions [41, 43]. Peripheral nerve injury initiates a tissular reaction distal to the damage site known as Wallerian degeneration that is followed by successful axonal regeneration [44, 45]. We studied the contribution of ERp57 to sciatic nerve degeneration and regeneration triggered by mechanical damage (Fig 4A). We confirmed the overexpression of ERp57 in the soma of sensory neurons in the dorsal root ganglia (DRG) and sciatic nerve using anti-FLAG immunohistochemistry (Fig 4B) or real-time PCR (S1B Fig). Of note, FLAG immunoreactivity in nerve preparations was observed in axons but also in Schwann cells (Fig 4B). To determine the possible modulation of ERp57 during peripheral nerve damage, the sciatic nerve was crushed and then the expression of ERp57 was analyzed by western blot after 12 or 24 hours post-injury (hpi), which corresponds to the time points when Wallerian degeneration is taking place [46–48]. Different nerve fragments were dissected including the medial region (M) containing the damage site, in addition to the proximal (P) and distal regions (D) (see schema in Fig 4A). The uninjured contralateral nerve was used as control (C). We observed an increase in ERp57 expression in the sciatic after nerve injury using western blot analysis that was higher in the medial region (Fig 4C).

To define the possible effects of ERp57 overexpression in motor control after nerve injury, animals were damaged in the right sciatic nerve and locomotor performance was evaluated using the sciatic nerve functional index (SFI) [49]. SFI analysis was measured over time until full motor recovery was observed (21 days post-injury (dpi)) [49]. Remarkably, a significant increase in locomotor function was observed in Tg-ERp57 mice at 9 and 14 dpi compared to Non-Tg mice (Fig 4D). Full recovery was observed in both groups of animals at 21 dpi. These results indicate that ERp57 overexpression in the nervous system accelerates locomotor function after peripheral nerve damage.

### ERp57 overexpression enhances axonal regeneration after peripheral nerve injury

**Fig 5:**
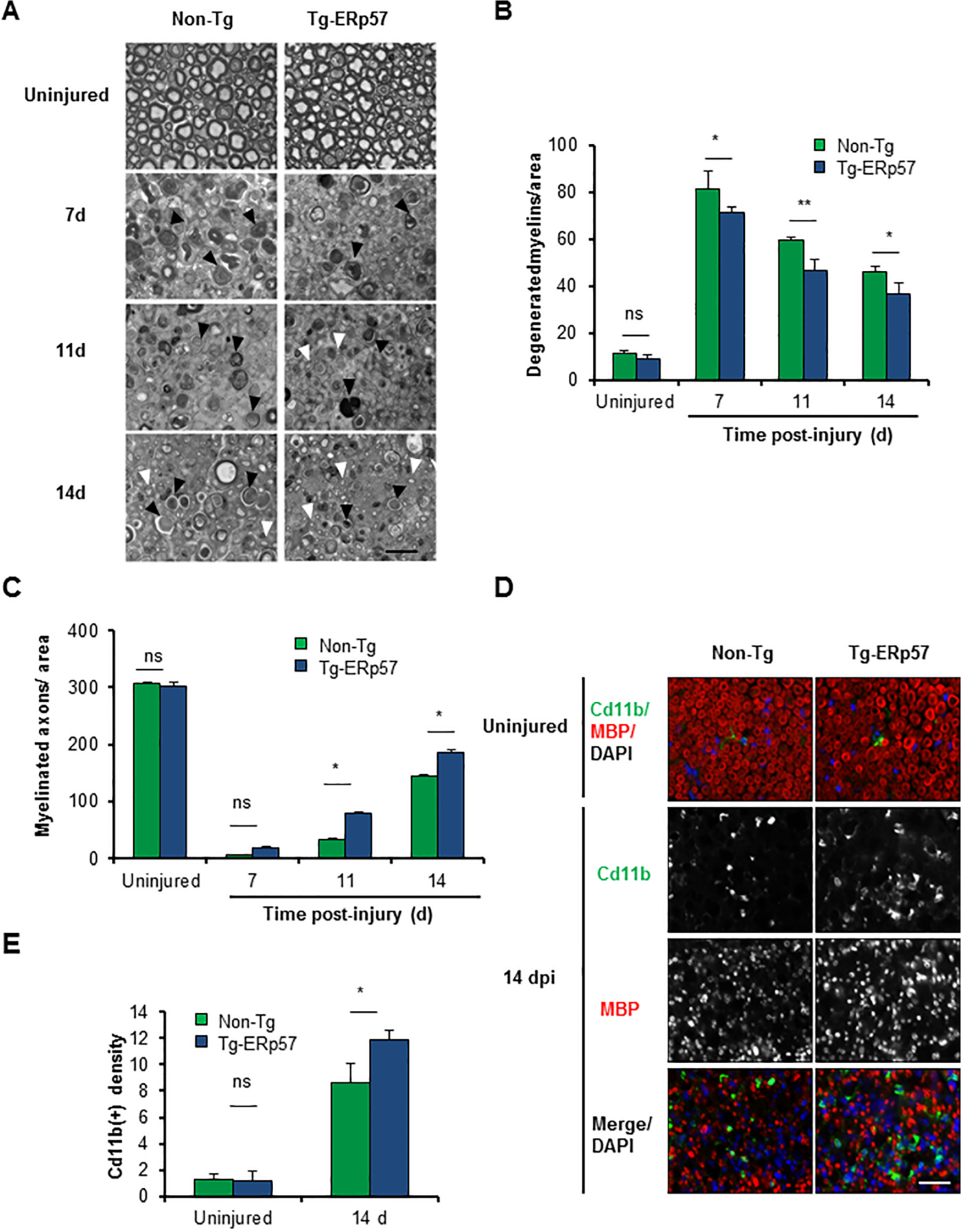
Overexpression of ERp57 in transgenic mice increases axonal regeneration after peripheral nerve injury. (A) Non-Tg and Tg-ERp57 mice were damaged and sciatic nerves were extracted at 7, 11 and 14 days post-injury (dpi). Transversal semi-thin sections were obtained from the distal region and remyelinated axons (white arrows) and degenerated myelins (black arrows) were analyzed. (B) Quantification of remyelinated axons and (C) degenerated myelin density was measured at 7, 11 and 14 days post-injury. (D) Tg- ERp57 and Non-Tg sciatic nerves were processed for immunofluorescence in uninjured conditions and at 14 dpi. Sciatic nerves were analyzed for MBP (red), Cd11b (green) and nuclei were stained using DAPI (blue). (E) The staining density for Cd11b was quantified 14 days after injury (right panel). Data is presented as mean ± S.E.M. * *p* < 0.05, ** *p* < 0.01, *** *p* < 0.001. For histological analysis, statistical differences were obtained using a student’s t-test (n = 3 animals per group). Scale bar: 20 μm.

We then evaluated myelin removal and regeneration of nerve fibers of Tg-ERp57 mice after sciatic nerve crush. Animals were injured and the morphology of myelinated axons was visualized and quantified in peripheral nerves at 3 mm distal from the injury site using semi-thin sections (Fig 5A). At basal levels, a homogeneous distribution of intact myelinated axons with condensed myelin was observed in both Tg-ERp57 and littermate control mice (Fig 5A, upper panel). Electron microscopy analysis also showed no differences in the ultrastructure of uninjured nerves (S4A Fig). We then quantified the content of myelinated and degenerating axons over time after sciatic nerve injury. In our crush model, an initial increase in degenerated myelin is observed that then decays progressively as myelin removal and axonal regeneration proceeds [44]. Consistent with the locomotor measurements, Tg-ERp57 mice exhibited a significant reduction in the number of degenerated myelin (Fig 5B), with a concomitant increase in the number of re-myelinated axons (Fig 5C). Qualitative electron microscopy analysis at 14 dpi corroborated these results (S4A Fig).

Myelin removal and subsequent axonal regeneration is mediated in part by Schwann cells and through the infiltration of macrophages at later stages after nerve injury [50, 51]. To analyze the impact of ERp57 overexpression on the cellular immune response associated to Wallerian degeneration, we evaluated the infiltration of Cd11b^+^-macrophages into injured nerves. Measurement of Cd11b^+^ density in the distal stump of Tg-ERp57 mice revealed a significant increase in macrophage content at 14 dpi when compared to Non-Tg littermates (Fig 5D and 5E). As control we monitored the levels of ERp57 in macrophages. Importantly, macrophages isolated from the lung of Tg-ERp57 animal (S4B Fig). Similarly, co-immunofluorescence analysis of FLAG-tagged ERp57 and the macrophage marker Cd11b did not show any co-expression in Tg-ERp57 mice at the sciatic nerve after peripheral nerve crush (S4C Fig). In contrast, a clear colocalization was observed between ERp57-FLAG and the Schwann cell marker MBP (S4D Fig). Taken together, these results indicate that overexpression of ERp57 modulates the axonal regeneration process, improving locomotor recovery after damage to the PNS.

## Discussion

PDIs are emerging as interesting therapeutic targets and biomarkers in multiple neurodegenerative diseases associated with protein misfolding and ER stress. However, so far no functional studies are available addressing the actual role of PDIs in neurodegeneration. ERp57 is a major PDI family member expressed in the nervous system and has been identified as one of the main proteins upregulated in tissue derived from ALS and Creutzfeldt-Jacob patients [15, 16, 18, 19, 22]. A few knockout mouse models for PDIs have been described to date, including ERp57, ERp29, ERdj5, PDIA1 and AGR2 [31, 52–57]. ERdj5 knockout mice develop altered ER proteostasis in salivary glands [58], whereas AGR2/PDIA17 knockout mice are viable, but show decreased levels of mucin, an essential component of the protective mucus in the intestine, resulting on severe colitis [56, 57]. Complete ERp57 deficiency in mice is lethal at embryonic day 13.5, possibly due to altered STAT3 signaling [59]. Conditional deletion of ERp57 in B cells leads to altered MHC class I peptide loading. However, no effects were detected on the secretion of immunoglobulins, a highly glycosylated protein containing disulfide bonds [52]. ERp29 deficiency alters the susceptibility of cells to apoptosis [53], whereas PDIA1 deficiency is lethal, and conditional deletion of this gene in platelets impairs thrombus formation [54]. Overall, in none of these studies CNS-related phenotypes were reported so far when the expression of PDIs was targeted *in vivo*.

Here we addressed the impact of ERp57 on pathological conditions affecting the nervous system by creating a transgenic mouse model. Tg-ERp57 animals presented increased locomotor recovery after peripheral nerve injury, associated with improved regeneration of axons and myelin removal. According with previous evidence, PNS damage triggers the upregulation of components of the ER proteostasis network such as calnexin, BiP and ERp29 [60, 61]. Consistent with this, we observed an upregulation of ERp57 levels in injured nerves. We speculate that our transgenic model may generate a preconditioned environment, enhancing the ability of neurons to handle protein-folding stress generated by the mechanical injury to the axon. Consistent with our results, a recent report described the occurrence of ER stress in models of peripheral nerve injury triggered by diabetes [62]. Treatment of mice with chemical chaperons reduced axonal degeneration, correlating with an attenuated ER stress response [62]. Importantly, another study identified the ER stress-related sensor LUMAN/CREB3 as an ER-located protein that is activated by mechanical injury to the axon [63]. LUMAN may communicate stress signals to the nucleus to enforce an adaptive reaction to reduce nerve damage.

In contrast to the protective consequences of ERp57 overexpression in the PNS, and despite expectations, our Tg-ERp57 mouse model did not show any protection on a pharmacological model of PD. Although ERp57 reduced the upregulation of *Pdia1* and *Erp72* mRNA levels after exposing animals to 6-OHDA, we were unable to detect any differential effects on dopaminergic neuron survival or striatal denervation in Tg-ERp57 animals. However, in our model we did not observe clear signs of UPR activation, suggesting that the modulation of ERp72 and PDIA1 expression by 6-OHDA may be ER stress-independent. Oxidative stress is the major contributor to 6-OHDA neurotoxicity [64, 65] and this condition has been associated with secondary proteostasis alterations in PD [66]. Oxidative conditions also result in ERp57 inactivation by S-nitrosylation, similarly to the effects reported for PDIA1 in human PD-derived tissue [24]. Thus, although we succeeded to overexpress ERp57 in the striatum and SNpc, it might be possible that ERp57 was post-translationally modified and inhibited by the oxidative stress generated by 6-OHDA, which may explain our negative results. It remains to be determined if ERp57 overexpression protects dopaminergic neurons on PD models based on alpha Synuclein aggregation.

ERp57 deficiency may result in impaired folding and function of myelin protein 0 (P0), one of the major peripheral myelin components [67]. The ERp57 binding partner, CNX, interacts and assists the folding of peripheral myelin proteins, contributing to peripheral neuropathies in mouse models [68]. CNX deficiency leads to myelinopathy associated with the occurrence of motor defects [28, 29]. Importantly, Schwann cells and oligodendrocytes are highly prone to undergo ER stress as demonstrated in many different disease models due to their high demand for protein and lipid synthesis [2, 69]. Since the PrP promoter is also active in Schwann cells [70, 71], we propose that ERp57 may have a relevant activity in sustaining glial proteostasis as a central component of the CNX-CRT cycle. In this context, ERp57 may contribute to maintain myelin structure or to reduce stress levels in Schwann cells. Previous evidence uncovered the consequences of altering Schwann cell proteostasis in the peripheral nerve. For example, *in vitro* studies using *Chop* deficient Schwann cells revealed an important proapoptotic activity of this factor upon pro-inflammatory challenges [72]. In the context of a demyelinating pathology, targeting PERK signaling in Charcot-Marie-Tooth-1B disease at the level of *Chop* [73] or eIF2 alpha phosphorylation provide strong protection [74, 75]. Taken together, our results describe a novel genetic model to manipulate ERp57 levels in the nervous system, demonstrating for the first time a relevant neuroprotective effect of this essential PDI in the PNS after mechanical injury.

## Materials and Methods

### Generation of the ERp57 transgenic mouse model

This study was carried out in strict accordance with the recommendations in the Guide for the Care and Use of Laboratory Animals of the National Institutes of Health. The protocol was approved by the Committee on the Ethics of Animal Experiments of the University of Chile (protocol number CBA#0265 FMUCH). Mice were housed under a 12:12 h light-dark cycle with access to food and water *ad libitum*.

The expression plasmid MoPrP.XhoI was kindly provided by David Borchelt of the Johns Hopkins School of Medicine, Baltimore, USA (Borchelt et al., 1996). Human ERp57 cDNA (Gene ID: 2923) was introduced into the MoPrP.XhoI plasmid and fused with the sequence coding for a FLAG tag using the XhoI restriction site. The resulting construct expresses FLAG-tag ERp57 at the N-terminus under the control of the prion (PrP) promoter. ERp57 transgenic mice were generated in the “Centro de Estudios Científicos” (CECS), Valdivia, Chile. The following primers were used for genotyping: *Erp57* 866 forward 5’-AATTCCTGGATGCTGGGCACAAAC -3’ and *Erp57* 1535 reverse 5’-TCTGCTTGTCATCGTCGTCCTTGT-3’. Briefly, 1 μL of DNA was incubated with 10 μL of Go Taq Master Mix (Promega) and 1 μM of each primer in a final volume of 20 μL. The PCR program used consisted in one cycle of denaturation for 5 min at 94°C, 20 cycles of 30 sec at 94°C, 30 sec at 65°C (decreasing 0.5°C per cycle), 50 sec at 72°C; 16 cycles of 50 sec at 94°C, 30 sec at 55°C, 50 sec at 72°C and a final cycle of elongation for 5 min at 72°C.

### Motor and behavioral tests

For the Rotarod test, mice were placed into a rod rotating (Model LE8500, Panlab SL) at an accelerating speed starting with 4 rpm up to 40 rpm within 2 min [76]. The time until mice could not maintain themselves on the rod was measured. Training was performed 3 times per day for 5 consecutive days. The test was applied once per week starting at 52 days of age until 80 days of age.

For the hanging test, individual mice were placed hanging with their forepaws on a horizontal bar (39 cm length and 35 cm height). The performance of the mouse and the body position was observed for 30 seconds and recorded with a video-camera. The test was performed 3 times in one day once per week starting at 53 days of age until 81 days. A score was derived from each video and averaged for the three measurements done at the same day as described before [77]: score 0 was given when the mouse could not hold onto the bar for more than 10 seconds; score 1, when the mouse maintained itself on the bar with the forelimbs for a minimum of 10 sec but not longer than 30 sec; score 2 if the mouse maintained itself with the forelimbs and completed 30 sec; score 3 if the mouse used the forelimbs and one or two of the hindlimbs but not the tail; score 4 if the mouse used all four limbs and the tail; and score 5 if the mouse actively escaped the bar in 30 sec.

### Pharmacological model of PD

Stereotaxic injections were performed in 3 month-old male and female Non-Tg and Tg-ERp57 mice. Mice were intraperitoneally anesthetized with ketamine/xylazine (ketamine 100 mg/kg, xylazine 10 mg/kg; Vetcom) and placed into a stereotaxic frame (David Kopf Instruments). Unilateral injections of 4 μg/μL 6-hydroxydopamine (6-OHDA; Sigma-Aldrich) dissolved in 0.2% ascorbic acid were administered as described before [38]. 6-OHDA injections were performed in a single point, injecting 2 μL in the right striatum using a 5 μL Hamilton syringe at the following coordinates: anteroposterior (AP) +0.07 cm; mediolateral (ML) −0.17 cm; dorsoventral (DV) −0.31 cm. For histological analysis mice were euthanized by CO_2_ narcosis 7 days after the surgical procedure.

The Cylinder test is designed to evaluate locomotor asymmetry in rodent models of PD. As the animal moves within an open-top, clear glass cylinder, its forelimb activity while rearing against the wall of the arena is recorded. Forelimb use is defined by the placement of the whole palm on the wall of the arena, which indicates its use for body support. Forelimb contacts while rearing were scored. The analysis was performed blinded. The result is plotted as the percentage of contralateral touches relative to total touches with both paws.

### Peripheral nerve crush model

Sciatic nerve injury was performed in 3-month-old male and female Non-Tg and Tg-ERp57 mice as described before [78]. Animals were intraperitoneally anesthetized with 2-2-2 tribromoethanol (330 mg/Kg, Sigma, St. Louis, MO, USA) and treated with Tramadol (30 mg/Kg) as analgesic. Surgical dissection of the skin and muscle was performed at the level of the sciatic notch to expose the right sciatic nerve. The sciatic nerve was crushed three times for 5 seconds with a Dumont #5 forceps (Fine Science Tools INC. CA, USA) and the skin was sutured with mouse metal clips. During recovery, mice were placed in a temperature-controlled chamber. At different days post-surgery, animals were euthanized by overdose of anesthesia and the sciatic nerve was removed for different analyses.

The sciatic nerve functional index (SFI) was performed to determine locomotor capability after sciatic nerve injury. Paw prints of individual mice were obtained by moistening the hindlimbs with black ink and having them walk unassisted along an 11 X 56 cm white paper corridor. Tracks were obtained before surgery (day 0), and 1, 3, 7, 9, 14 and 21 days after nerve injury. All paw prints were obtained and analyzed in a blinded fashion. Two different parameters were analyzed: toe spread (TS), the distance between the first and fifth toes, and print length (PL), the distance between the third toe and the hind pad. Measurements of all parameters were made for the right injured hindpaw (experimental; E) and the left uninjured hindpaw (normal; N) and the SFI was calculated according to the following formula: SFI = 11.89 ((ETS–NTS) / NTS) – 51.2 ((EPL–NPL) / NPL) –7.5 as previously described [79].

### Tissue extract

The ventral midbrain (containing the entire Substancia Nigra pars-compacta, SNpc), striatum, and cortex from both hemispheres were dissected and homogenized in 100 μL of ice-cold 0.1 M PBS (pH 7.4) supplemented with a protease inhibitor cocktail (Roche). The homogenate was divided into two fractions for total mRNA and protein extraction, followed by standard purification and quantification protocols [80]. Protein extraction was performed in RIPA buffer (20 mM Tris pH 8.0, 150 mM NaCl, 0.1% SDS, 0.5% deoxycholate, and 0.5% Triton X-100) containing a protease inhibitor cocktail and phosphatase inhibitor cocktail (Sigma-Aldrich).

For sciatic nerves extracts, the nerve segments containing the injury region (5 mm of medial region), and proximal and distal nerve segments of the same size were collected. The contralateral sciatic nerve was used as a control. Sciatic nerves were homogenized in extraction buffer (95 mM NaCl, 25 mM Tris-HCl pH 7.4, 10 mM EDTA pH 8.0, 1% SDS, 1 mM NaF, 1 mM Na_3_VO_4_ and 1% Protease Inhibitor Cocktail [PIC, Sigma-Aldrich, #P8340]). Lysates were sonicated, centrifuged at 13,000 rpm for 10 min at 4°C and the supernatants were used for protein analysis. Samples were loaded onto SDS/PAGE gels and blotted onto PVDF membranes. The following antibodies and dilutions were used: anti-HSP90, (1:5000, sc-7947, H114, Santa Cruz, Santa Cruz, CA, USA); anti-ERp57 (1:3000, SPA-585, Stressgen). Band intensities were quantified and normalized to Hsp90 as a loading control. Densitometry analysis was performed using ImageJ software (NIH, Bethesda, MD, USA).

### RNA extraction and real time PCR

Total RNA was isolated from ventral midbrain (containing entire SNpc), dorsal root ganglia (from lumbar vertebrae L3 and L4) and medial sciatic nerves. After homogenization in PBS, total mRNA was purified using TRIzol (Life Technologies), and cDNA was synthesized from 1 μg of RNA using an Applied Biosystems Reverse-Transcription Kit. Quantitative real-time PCR was performed in a Stratagene lightcycler system using SYBR Green (Applied Biosystems) using the following primers: *Erp72* forward, 5′-ACTCTCCGGGAATTTGTCACA-3′; *Erp72* reverse, 5′-ATGTCGTTGGCGAGTAGCATC-3′; *Pdia1* forward, 5’-CAAGATCAAGCCCCACCTGAT-3’; *Pdia1* reverse, 5’-AGTTCGCCCCAACCAGTACTT-3’;-*human Erp57* forward, 5’- GCC TCC GAC GTG CTA GAA C -3’; *human Erp57* reverse 5’- GCG AAG AAC TCG ACG AGC AT -3’; *Chop* forward 5’-TGGAGAGCGAGGGCTTTG-3’, *Chop* reverse 5’-GTCCCTAGCTTGGCTGACAGA-3’.

*Xbp1* mRNA splicing assay was performed as previously described [81] using the following primers: *Xbp1* forward 5’-ACACGCTTGGGAATGGACAC-3’; *Xbp1* reverse: 5’-CCATGGAAGATGTTCTGGG-3’.

### Histological analysis

For ERp57-FLAG expression analysis, Tg-ERp57 and Non-Tg control littermates were anesthetized and perfused through the ascending aorta with isotonic saline, followed by ice-cold 4% paraformaldehyde in 0.1 M PBS (pH 7.4). Tissue was sectioned using a Leica cryostat (Leica, Nussloch, Germany) as follow: brain, 25 μm coronal sections; spinal cord, 18 μm transversal sections; and dorsal root ganglia (DRG) and sciatic nerve, 10 μm transversal sections. For immunohystochemistry, tissue was incubated 30 min at room temperature (RT) with 3% H_2_O_2_ in 10% methanol in PBS, followed by epitope retrieval using 10 mM citrate buffer pH 6.0 (home made) for 15 min at 95°C. After overnight (ON) incubation in blocking solution (BSA 5%, Triton X-100 0.3% in PBS) at 4°C, sections were incubated with rabbit anti-FLAG antibody (1:250, F7245, Sigma) for 2 h at RT, followed by incubation with HRP-conjugated goat anti-rabbit antibody (1:1000, Invitrogen) for 1 h incubation at RT. Immunoreactivity was developed using DAB HRP substrate kit (Vector Laboratories). Tissue staining was visualized with an inverted microscope (Olympus IX71). For co-immunofluorescence analysis, tissue was incubated with 10 mM citrate buffer pH 6.0 (home made) for 15 min at 95°C for epitope retrieval. After 1 h in blocking solution (5% BSA, 0.3% Triton X-100 in PBS) at RT, sections were incubated with rabbit anti-FLAG antibody (1:250, F7245, Sigma) for 2 h at RT, followed by incubation with mouse anti-TH (1:300,Millipore) or anti-GFAP (1:300, Sigma) ON at RT. Slices were then incubated with goat anti-rabbit (1:1000, Alexa 568, Invitrogen) and goat anti-mouse antibody (1:1000, Alexa 488, Invitrogen) for 1 h incubation at RT. Slices were visualized with a confocal microscope (Olympus Spectral FV1000 confocal microscope).

To analyze CHOP expression, tissue was incubated 30 min at room temperature with 3% H_2_O_2_ in 10% methanol in PBS, followed by epitope retrieval using buffer citrate pH 6.0 (DAKO) for 20 min at 95°C. After incubation in blocking solution (10% Goat serum, 4% BSA, 0.1% Triton X-100 in PBS) for 1.5 h at RT, sections were incubated with rabbit anti-CHOP antibody (1:100, Santa Cruz) ON at 4°C, followed by incubation with HRP-conjugated goat anti-rabbit antibody (1:1000, Invitrogen) for 1 h incubation at RT. Immunoreactivity was developed using DAB HRP substrate kit (Vector Laboratories). Tissue staining was visualized with an inverted microscope (Olympus IX71).

For the quantification of dopaminergic neuron degeneration in the 6-OHDA model, the brain was sectioned into 25 μm coronal frozen sections containing the rostral striatum and midbrain on a Leica cryostat (Leica, Nussloch, Germany). Free-floating midbrain and striatal tissue sections were obtained and processed for immunohistochemistry. For immunohistochemical analysis, sections were incubated overnight at 4°C in blocking solution with tyrosine hydroxylase (TH) (1:2500; Calbiochem) primary antibody and developed with biotinylated secondary anti-rabbit antibody (1:500; Vector Laboratories) and avidin-biotin peroxidase complex (ABC Elite Kit; Vector Laboratories). Tissue was visualized with an inverted microscope (Olympus IX71).

Sciatic nerves were extracted at 14 days post injury (dpi). A 3 mm segment located 3 mm distal to the crush site was removed and fixed for 1 h in 4% paraformaldehyde in 0.1 M PBS (pH 7.4). Nerves were then subjected to a sucrose gradient (10, 20 and 30% sucrose in PBS), included in optimal cutting temperature compound (OCT, Sakura Finetek) and fast frozen in liquid nitrogen. The tissue was transversally sectioned (10 μm thickness) using a cryostat (Leica, Nussloch, Germany) and mounted on Superfrost Plus slides (Thermo Fisher Scientific). For immunofluorescence, sections were blocked/permeabilized with 2% fish skin gelatin (Sigma-Aldrich) and 0.1% Triton X-100 in PBS for 1 h at RT and incubated with primary antibodies in the same solution overnight at 4°C. Then, incubated in secondary antibodies for 2 h at RT and mounted in Vectashield (Vector Laboratories) as previously described [82]. Sections were immunostained using the following antibodies and dilutions: rabbit anti-myelin basic protein (MBP) (1:500, M3821, Sigma), rat anti-MBP (1:50, a gift from ML Feltri, Hunter James Kelly Research Institute & Biochemistry, University at Buffalo, New York, USA), rat anti-Cd11b (1:500, MCA74G, Serotec) and rabbit anti-FLAG (1:250, F7245, Sigma). Electron microscopy analyses in sciatic nerves were made at 7, 11 and 14 dpi. A 3 mm region of the sciatic nerve, located 6 mm distal to the injury site was removed and fixed overnight with 2.5% glutaraldehyde, 0.01% picric acid and 0.1 M cacodylate buffer, pH 7.4. Nerves were incubated in the same buffer with 1% OsO_4_ for 1 h and then immersed in 2% uranyl acetate for 2h, dehydrated in a gradient of ethanol and propylene oxide and infiltrated in Epon (Ted Pella) as previously described [83]. Transversal semi-thin and ultra-thin sections were obtained using an ultramicrotome. All images of tissue sections were obtained in an inverted Olympus fluorescent microscope and analyzed using ImageJ software (NIH, Bethesda, MD, USA).

### Dopaminergic neuron counting

Estimation of the number of TH-positive neurons stained by immunohistochemistry was performed manually. Results are expressed as the total number of TH-positive neurons per hemisphere. To determine the percentage of TH-positive cell loss in the SNpc of 6-OHDA injected mice, the number of dopaminergic cells in the injected and non-injected side was determined by counting in a blinded manner the total number of TH-positive cells in midbrain serial sections containing the entire SNpc (between the AP−0.29 and AP−0.35 cm coordinates). Results were expressed as the percentage of TH-positive neurons in the injected hemisphere compared with the non-injected side. In addition, striatal denervation was quantified by measuring the optic density from serial sections covering the entire striatum using ImageJ software (NIH, Bethesda, MD, USA). The total integrated density per hemisphere was quantified. Results are expressed as the percentage of the integrated density in the injected hemisphere compared to the non-injected side.

### Isolation of alveolar macrophages

Lungs were minced and then were digested for 30 min at 37°C with Collagenase (1 mg/ml; Roche) and recombinant DNase I (0.01 U/μL) without serum, followed by lysis of red blood cells. Samples were stained with allophycocyanin anti-CD11c (N418; BD Pharmingen), allophycocyanin-indotricarbocyanine–conjugated antibody to MHC class II (M5/114, 15, 2; Biolegend), 7-aminoactinomycin D (7-AAD) viability dye (Life technologies) and Fc Block antibody (2.4G2, BD Pharmingen). Alveolar macrophages were isolated by cell sorting as reported [84]. Briefly, cells were isolated with gating on live cells, FSC/SSC high granularity, CD11c+, MHCII intermediate and high auto fluorescent signal. Cells were sorted on a FACSAria (BD Biosciences).

### Models of ER stress

SH-SY5Y cells were cultured in DMEM supplemented with 10% fetal bovine serum and antibiotics (10,000 U/ml Penicillin, 10 μg/ml streptomycin), at 37°C and 5% CO_2_. For induction of ER stress *in vitro*, the ER stressors tunicamycin, was added to the cell culture medium of SH-SY5Y cells followed by the measurement of ER stress markers by PCR. For induction of ER stress *in vivo*, mice received a single intracerebral injection of 2 μL of tunicamycin (5 5 μg/ μL) diluted in DMSO at the following coordinates: AP: -0,29 cm ML: -0,13 cm and DV: -0,42 cm and sacrificed 24 h post injection.

## Supporting Information

S1 Fig Generation of a human ERp57-FLAG transgenic mice. (A, left panel) ERp57 protein levels were analyzed in the substancia nigra of ERp57 transgenic (Tg-ERp57) mice (n = 5) and littermate control non-transgenic (Non-Tg) animals (n = 6) using Western blot. β-Actin was used as a loading control. (A, right Panel) quantification of expression levels normalized against Non-Tg protein levels. (B) *hERp57* mRNA levels were determined in dissected dorsal root ganglia (DRG) (left) and sciatic nerve (right) of Non-Tg (n = 3) and Tg-ERp57 (n = 5) mice using real-time PCR. (C) Co-immunofluorescence anti-FLAG(red) and anti-TH(green) in midbrain tissue (upper panel) and anti-FLAG (red) and anti-GFAP (green) in hippocampus (bottom panel), showing co-localization of ERp57-FLAG transgene and TH dopaminergic neuron marker (yellow) but not co-localization with GFAP astrocyte marker. Scale bar: 50 μm. In A and B data are shown as mean ± S.E.M. * *p* < 0.05, ** *p* < 0.01, *** *p* < 0.001.
(TIF)

S2 Fig The 6-OHDA model. Immunohistochemistry anti-TH in serial sections of 25 μm of thick spaced by 100 μm covering the entire substantia nigra (A) and striatum (B) of one representative Non-Tg and Tg-ERp57 animal injected with 8 μg of 6-OHDA into the right striatum and sacrificed 7 days later.
(TIF)

S3 Fig Lack of an ER stress in the SNpc of animals exposed to 6-OHDA. (A) Analysis of Chop expression levels using immunohistochemistry of animals injected with 8 μg of 6-OHDA into the right striatum and sacrificed 7 days later (upper panel). The image is representative of Non-Tg and Tg-ERp57 (n = 3 per group). As control to induce Chop, animals were injected with tunicamycin (Tm) and sacrificed 24 h later (bottom panel). Scale bar: 200 μm. (B) C*hop* mRNA levels were determined by real-time PCR in dissected SNpc from WT animals injected with 8 μg de 6-OHDA after indicated time post injection. As positive animals were injected with 10 μg of Tm directly into the SNpc and sacrificed 24 h post-injection. Values were normalized by actin and are shown as fold of induction relative to non-injected side. (C) *Xbp1* mRNA splicing was monitored in the SNpc or the striatum (D) of animals injected with 6-OHDA for indicated time points. The injected and non-injected side of the same animal was compared in two independent animals per group. As positive control for the assay, MEFs cells treated with 2,5 μg/ml of Tm for 16 h. (D) *Xbp1* mRNA splicing was also monitored in SH-SY5Y cells treated with 6-OHDA for indicated time points and concentrations. NT: not treated SH-SY5Y cells, (-) negative control without template, *Xbp1u*: unspliced *Xbp1* mRNA, *Xbp1s*: spliced *Xbp1* mRNA.
(TIF)

S4 Fig ERp57 overexpression reduces axonal degeneration. (A) Electron microscopy of Non-Tg and Tg-ERp57 uninjured and 14 days-damaged nerves. In uninjured conditions white arrowheads indicate axoplasm of myelinated fibers, black arrowheads, compact myelin sheaths and asterisks, unmyelinated fibers. At 14 days post-injury black arrows indicated degenerated myelins and white arrows, remyelinated axons. Scale bar: 4 μm. (B) *hERp57* mRNA levels were determined in macrophages isolated from alveoli of Non-Tg and Tg-ERp57 mice after cell sorting of Cd11b-positive cells using real-time PCR (n = 2 per group). Cortex tissue from these animals was used as positive control. (C) Non-Tg and Tg-ERp57 mice were damaged and sciatic nerves were extracted at 14 days post-injury. Contralateral uninjured nerves were used as control. Transversal slides were processed for immunofluorescence for FLAG (red), MBP (green) and DAPI (blue) to identify Schwann cells. (D) Animals described in C were used for immunofluorescence to stain FLAG (red), Cd11b (green) and DAPI (blue) to analyse the infiltrating macrophage population. At the right panels, magnifications of indicated areas are shown. Scale bar: 20 mm. In B data are shown as mean.
(TIF)
