# Correction: Functional Role of the Disulfide Isomerase ERp57 in Axonal Regeneration

**DOI:** 10.1371/journal.pone.0140200

**Published:** 2015-10-02

**Authors:** Valentina Castillo, Maritza Oñate, Ute Woehlbier, Pablo Rozas, Catherine Andreu, Danilo Medinas, Pamela Valdés, Fabiola Osorio, Gabriela Mercado, René L. Vidal, Bredford Kerr, Felipe A. Court, Claudio Hetz

The following information is missing from the Funding section: The Centro de Estudios Científicos CECs is funded by the Centers of Excellence Base Financing Program of CONICYT.
