# Supplementary figures and images for: Functional Role of the Disulfide Isomerase ERp57 in Axonal Regeneration
Source: PLoS One. 2015 Sep 11;10(9):e0136620. doi: 10.1371/journal.pone.0136620 (PMC4567344; doi:10.1371/journal.pone.0136620)

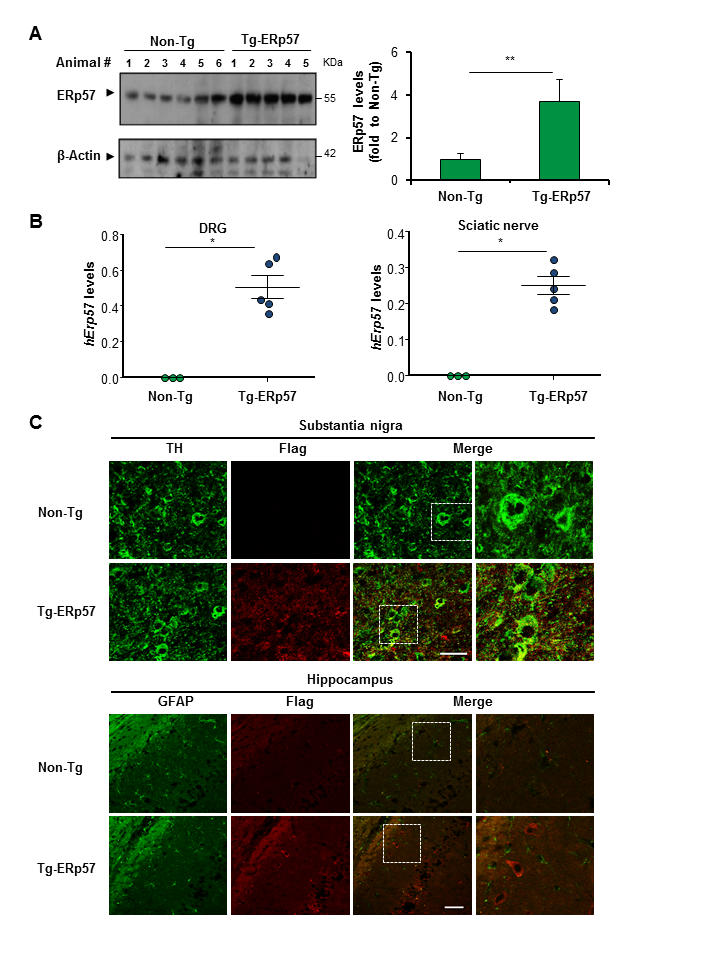

Supplement: S1 Fig — (A, left panel) ERp57 protein levels were analyzed in the substancia nigra of ERp57 transgenic (Tg-ERp57) mice (n = 5) and littermate control non-transgenic (Non-Tg) animals (n = 6) using Western blot. β-Actin was used as a loading control. (A, right Panel) quantification of expression levels normalized against Non-Tg protein levels. (B) hERp57 mRNA levels were determined in dissected dorsal root ganglia (DRG) (left) and sciatic nerve (right) of Non-Tg (n = 3) and Tg-ERp57 (n = 5) mice using real-time PCR. (C) Co-immunofluorescence anti-FLAG(red) and anti-TH(green) in midbrain tissue (upper panel) and anti-FLAG (red) and anti-GFAP (green) in hippocampus (bottom panel), showing co-localization of ERp57-FLAG transgene and TH dopaminergic neuron marker (yellow) but not co-localization with GFAP astrocyte marker. Scale bar: 50 μm. In A and B data are shown as mean ± S.E.M. * p < 0.05, ** p < 0.01, *** p < 0.001. (TIF) [file pone.0136620.s001.tif]

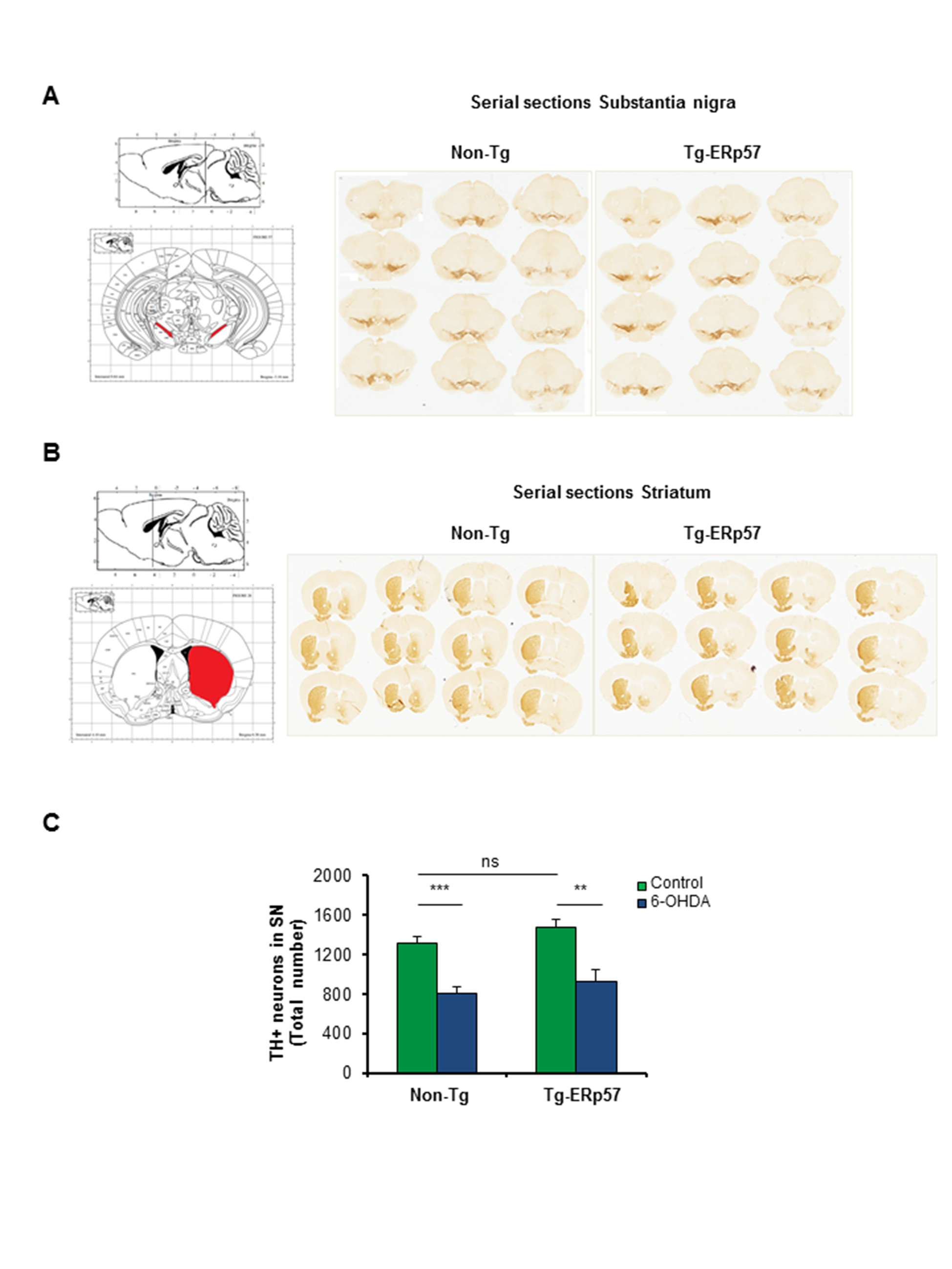

Supplement: S2 Fig — Immunohistochemistry anti-TH in serial sections of 25 μm of thick spaced by 100 μm covering the entire substantia nigra (A) and striatum (B) of one representative Non-Tg and Tg-ERp57 animal injected with 8 μg of 6-OHDA into the right striatum and sacrificed 7 days later. (TIF) [file pone.0136620.s002.tif]

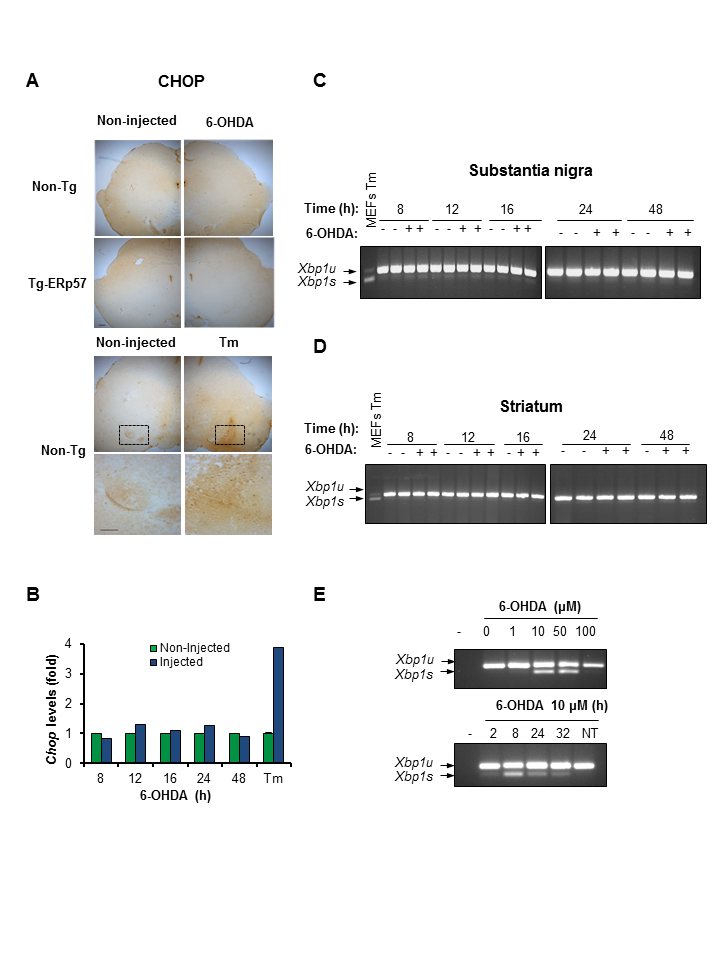

Supplement: S3 Fig — (A) Analysis of Chop expression levels using immunohistochemistry of animals injected with 8 μg of 6-OHDA into the right striatum and sacrificed 7 days later (upper panel). The image is representative of Non-Tg and Tg-ERp57 (n = 3 per group). As control to induce Chop, animals were injected with tunicamycin (Tm) and sacrificed 24 h later (bottom panel). Scale bar: 200 μm. (B) Chop mRNA levels were determined by real-time PCR in dissected SNpc from WT animals injected with 8 μg de 6-OHDA after indicated time post injection. As positive animals were injected with 10 μg of Tm directly into the SNpc and sacrificed 24 h post-injection. Values were normalized by actin and are shown as fold of induction relative to non-injected side. (C) Xbp1 mRNA splicing was monitored in the SNpc or the striatum (D) of animals injected with 6-OHDA for indicated time points. The injected and non-injected side of the same animal was compared in two independent animals per group. As positive control for the assay, MEFs cells treated with 2,5 μg/ml of Tm for 16 h. (D) Xbp1 mRNA splicing was also monitored in SH-SY5Y cells treated with 6-OHDA for indicated time points and concentrations. NT: not treated SH-SY5Y cells, (-) negative control without template, Xbp1u: unspliced Xbp1 mRNA, Xbp1s: spliced Xbp1 mRNA. (TIF) [file pone.0136620.s003.tif]

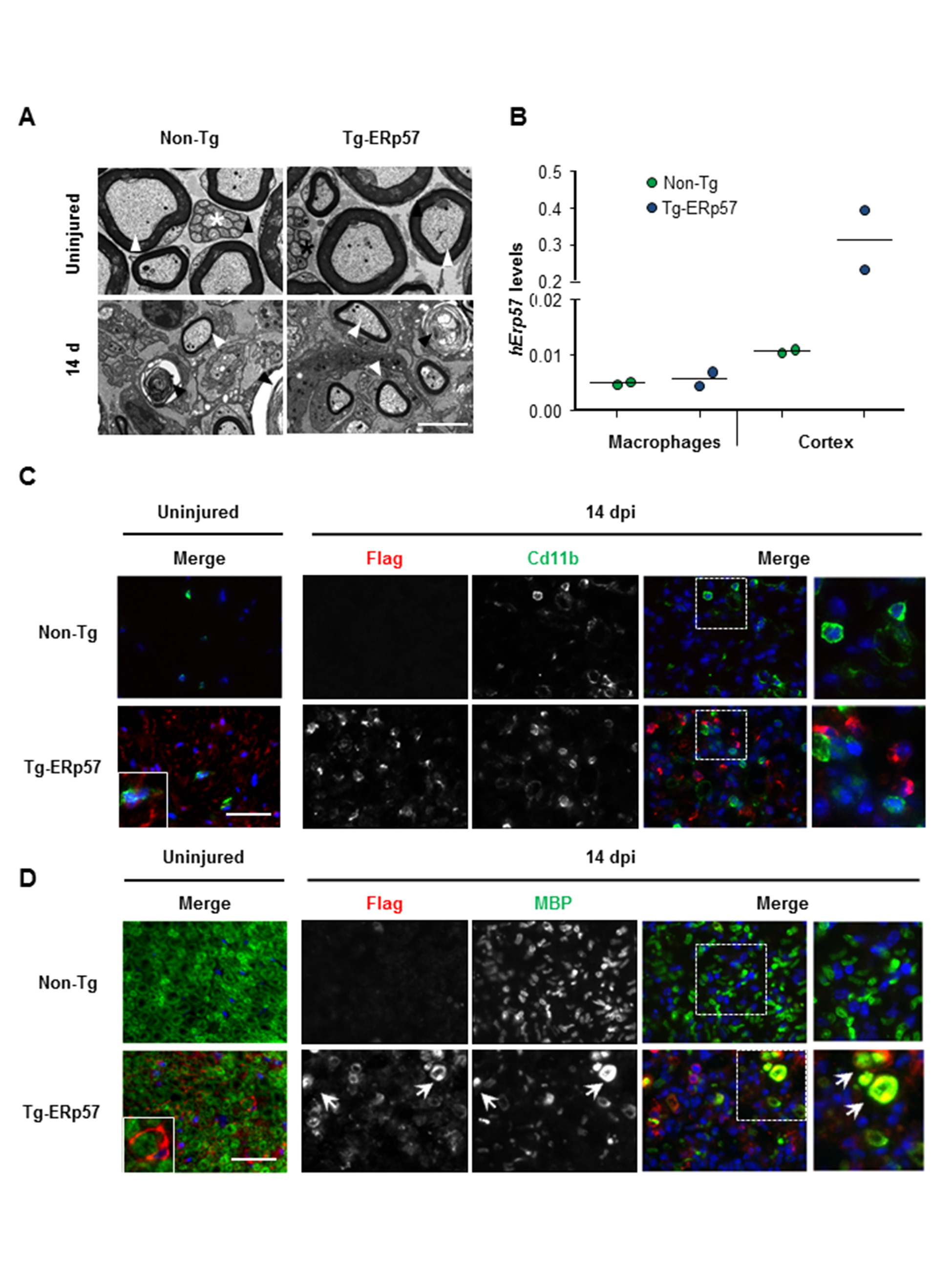

Supplement: S4 Fig — (A) Electron microscopy of Non-Tg and Tg-ERp57 uninjured and 14 days-damaged nerves. In uninjured conditions white arrowheads indicate axoplasm of myelinated fibers, black arrowheads, compact myelin sheaths and asterisks, unmyelinated fibers. At 14 days post-injury black arrows indicated degenerated myelins and white arrows, remyelinated axons. Scale bar: 4 μm. (B) hERp57 mRNA levels were determined in macrophages isolated from alveoli of Non-Tg and Tg-ERp57 mice after cell sorting of Cd11b-positive cells using real-time PCR (n = 2 per group). Cortex tissue from these animals was used as positive control. (C) Non-Tg and Tg-ERp57 mice were damaged and sciatic nerves were extracted at 14 days post-injury. Contralateral uninjured nerves were used as control. Transversal slides were processed for immunofluorescence for FLAG (red), MBP (green) and DAPI (blue) to identify Schwann cells. (D) Animals described in C were used for immunofluorescence to stain FLAG (red), Cd11b (green) and DAPI (blue) to analyse the infiltrating macrophage population. At the right panels, magnifications of indicated areas are shown. Scale bar: 20 mm. In B data are shown as mean. (TIF) [file pone.0136620.s004.tif]
